# New Insights Into Monogenic Causes of Osteoporosis

**DOI:** 10.3389/fendo.2019.00070

**Published:** 2019-02-25

**Authors:** Riikka E. Mäkitie, Alice Costantini, Anders Kämpe, Jessica J. Alm, Outi Mäkitie

**Affiliations:** ^1^Folkhälsan Institute of Genetics and University of Helsinki, Helsinki, Finland; ^2^Research Program for Clinical and Molecular Metabolism, Faculty of Medicine, University of Helsinki, Helsinki, Finland; ^3^Department of Molecular Medicine and Surgery and Center for Molecular Medicine, Karolinska Institutet, Stockholm, Sweden; ^4^Children's Hospital, Pediatric Research Center, University of Helsinki and HUS Helsinki University Hospital, Helsinki, Finland; ^5^Clinical Genetics, Karolinska University Hospital, Stockholm, Sweden

**Keywords:** early-onset osteoporosis, Wnt signaling, osteogenesis imperfecta, PLS3, bone metabolism

## Abstract

Osteoporosis, characterized by deteriorated bone microarchitecture and low bone mineral density, is a chronic skeletal disease with high worldwide prevalence. Osteoporosis related to aging is the most common form and causes significant morbidity and mortality. Rare, monogenic forms of osteoporosis have their onset usually in childhood or young adulthood and have specific phenotypic features and clinical course depending on the underlying cause. The most common form is osteogenesis imperfecta linked to mutations in *COL1A1* and *COL1A2*, the two genes encoding type I collagen. However, in the past years, remarkable advancements in bone research have expanded our understanding of the intricacies behind bone metabolism and identified novel molecular mechanisms contributing to skeletal health and disease. Especially high-throughput sequencing techniques have made family-based studies an efficient way to identify single genes causative of rare monogenic forms of osteoporosis and these have yielded several novel genes that encode proteins partaking in type I collagen modification or regulating bone cell function directly. New forms of monogenic osteoporosis, such as autosomal dominant osteoporosis caused by *WNT1* mutations or X-linked osteoporosis due to *PLS3* mutations, have revealed previously unidentified bone-regulating proteins and clarified specific roles of bone cells, expanded our understanding of possible inheritance mechanisms and paces of disease progression, and highlighted the potential of monogenic bone diseases to extend beyond the skeletal tissue. The novel gene discoveries have introduced new challenges to the classification and diagnosis of monogenic osteoporosis, but also provided promising new molecular targets for development of pharmacotherapies. In this article we give an overview of the recent discoveries in the area of monogenic forms of osteoporosis, describing the key cellular mechanisms leading to skeletal fragility, the major recent research findings and the essential challenges and avenues in future diagnostics and treatments.

## Introduction

### Bone Health

Bone is a rigid connective tissue composed mainly of organic components (90% type I collagen, the rest other non-collagenous structural proteins and cells) and inorganic minerals (mostly calcium hydroxyapatite). These combined give bones their sturdiness to withstand an individual's weight and the elasticity to enable movement and resist fractures. Bone comprises dense and compact cortical bone and cancellous, loosely-webbed trabecular bone, and serves as a reservoir for minerals, growth factors, cytokines, and fat. Bone also functions as an endocrine organ by secreting several systemic hormonal factors (1).

Bone is all but a quiescent tissue—it undergoes active renewing and remodeling throughout life. By coupled, successive processes of bone resorption and bone formation, together called “bone turnover,” old or damaged bone is eroded and replaced by new bone to maintain healthy and strong bone tissue. Throughout childhood and adolescent growth, the period of bone mass accrual, bone turnover is formation-favoring, until the highest amount of bone mass, termed “peak bone mass,” is attained by young adulthood. Thereafter, bone mass remains fairly constant until bone resorption begins to dominate by the age of menopause and bone mass slowly declines.

Factors that impede skeletal growth in childhood or accelerate bone loss later in adulthood, such as long-term or chronic illnesses, glucocorticoid treatment and other medications, hypogonadism and menopause, other endocrine disorders and cancers, impose a great risk for low bone mass and osteoporosis (1–3). In childhood, especially glucocorticoids play a major role in secondary osteoporosis. Studies on patients receiving systemic steroids for acute lymphoblastic leukemia (4), juvenile idiopathic arthritis (5, 6), Duchenne muscular dystrophy (7) or asthma (8) all indicate increased peripheral and vertebral fracture rates.

### Osteoporosis

Osteoporosis is a chronic skeletal disease with high prevalence and mortality worldwide. It is characterized by low bone mass and bone mineral density (BMD), and by destructed bone microarchitecture that often results from imbalanced bone formation and resorption or from abnormal matrix. Impaired bone quality leads to compromised bone strength and high propensity to low-energy fractures in long bones and vertebrae (9). Osteoporosis, with frequent fractures, pain and physical limitations, causes significant human suffering and burdens the health care system (9). BMD is considered to define osteoporosis and risk of fractures. It is assessed using dual-energy X-ray absorptiometry (DXA), where reduction of more than 2.5 standard deviations from the normal mean for young adults (T-score) is diagnostic of osteoporosis. Of note, osteopenia (T-score 1.0 to −2.5) together with a high probability of fractures, or a fragility fracture without another metabolic bone disease and independent of BMD are also clinically indicative of osteoporosis. Pediatric osteoporosis requires more than mere DXA-determined low BMD, as variation in growth and pubertal maturation make interpretation of BMD values challenging. Therefore, age-, gender-, and body size–adjusted DXA measurements (Z-scores) must be considered together with fracture history. A pathologic fracture history entails (i) ≥2 clinically significant long bone fractures by age 10 years, (ii) ≥3 clinically significant long bone fractures by 19 years, or (iii) one or more vertebral compression fractures in the absence of high-energy trauma, meaning a ≥20% loss in vertebral anterior, middle or posterior height. However, a vertebral compression fracture alone suffices for the diagnosis of pediatric osteoporosis even in the presence of normal BMD (2, 9–12).

## Genetics in Bone Health

### Genetics in Bone Health

Genetics play a substantial role in determining an individual's skeletal strength, bone microarchitectural properties and risk of osteoporosis. BMD is known to be a highly heritable trait and twin studies have shown genetic factors to determine up to 80% of its variance (13, 14). Genetic factors influence bone health in a polygenic manner and multiple gene variants, or single nucleotide polymorphisms (SNPs), in several different genes each contribute to the overall risk for compromised bone health. Recent research, especially large-scale genome-wide association studies in large cohorts, has elucidated the complexity of genetic networks that are important for bone metabolism but also evidenced limitations in our current knowledge. On the other hand, significant scientific advances have been made by studying rare monogenic forms of osteoporosis in which one mutation in a single gene with a major role in bone metabolism dominates and is alone sufficient to cause osteoporosis. Technical advancements in research methods, especially high through-put sequencing techniques, have made family-based studies an efficient way to identify new genes relevant to osteoporosis. Such studies have enabled recognition of novel molecular mechanisms and given leeway to understanding the intricacies behind bone metabolism (13, 14). In this article we only briefly summarize GWAS methodology and recent advancements while the main focus is on discoveries made from family-based research on patients and families with monogenic forms of osteoporosis.

### Genome Wide Studies to Identify Contributing Genetic Factors

**Figure 1:**
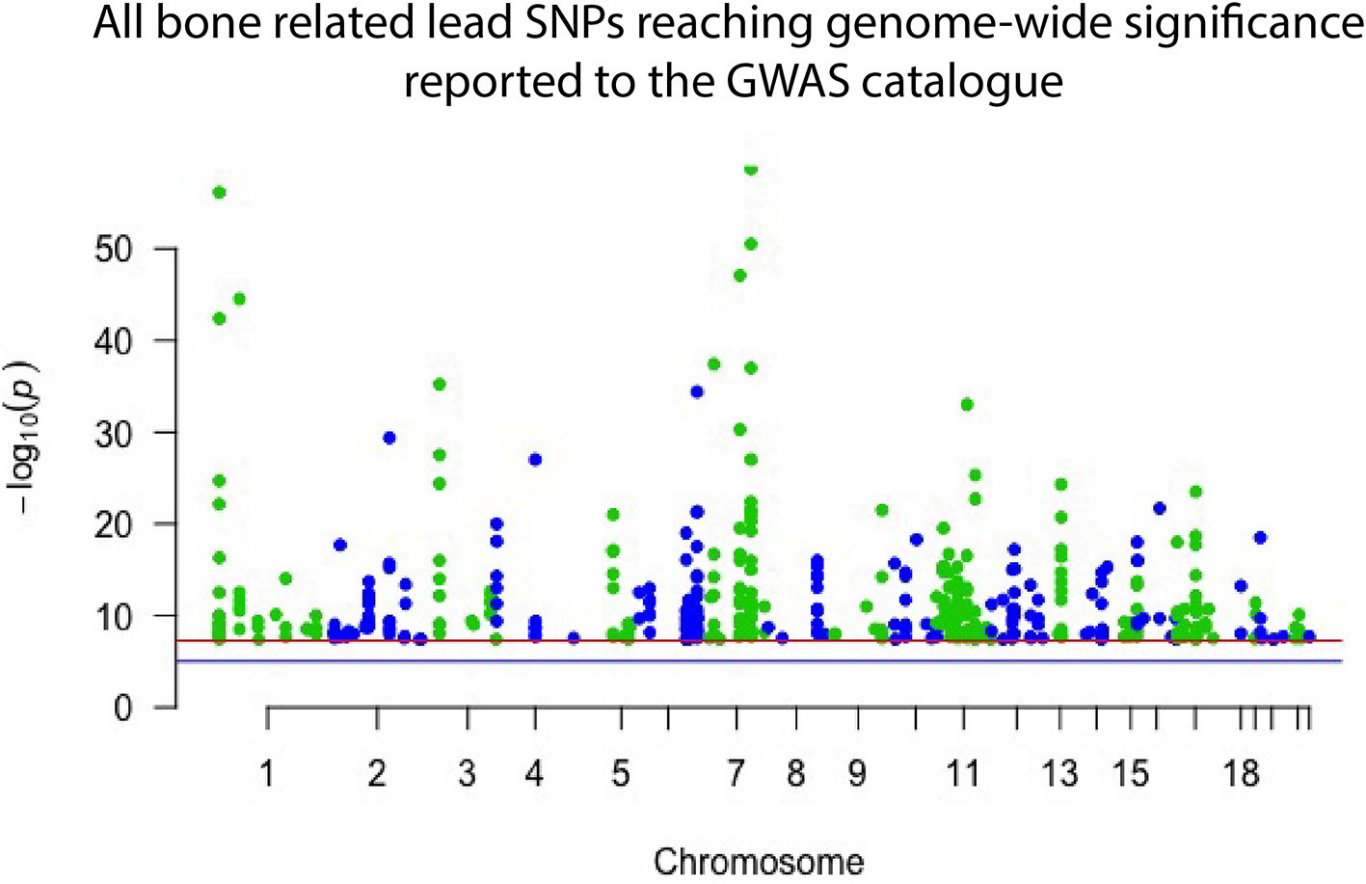
Manhattan plot displaying all lead SNPs independently associated with bone-related traits reported to the GWAS catalog as of September 2018. The associated SNPs highlight genomic regions important to bone. However, they each have only a minor effect on an individual's skeletal qualities and risk of osteoporosis and hence have limited use in clinical practice.

Genome wide association studies (GWASs) have proven successful and robust in deciphering the genetic mechanisms underlying complex diseases, including osteoporosis (14, 15). As mentioned, single nucleotide variants (SNVs) in several different genomic sites all contribute to bone quality and strength and risk of osteoporosis but are often very common in the general population and, by themselves, have only a minor effect (16). The current GWAS catalog, released in September 2018, comprises 55 separate studies focusing on bone properties, fractures or osteoporosis. Together they report 425 different lead SNVs, in 118 different genomic regions, that associate with some aspect of bone on a genome-wide significant level (Figure 1). From these, altogether 144 different genes are reported to be directly linked to, or plausible candidate effectors, for the identified signals. Of note, this catalog is not entirely up to date due to the extensive curation required before publication in the GWAS catalog. Recently, Kemp et al. undertook a colossal genome-wide search for genetic factors correlating with BMD, estimated from quantitative ultrasound of the heel (eBMD) (17). The GWAS is the largest to date, encompassing a total of 142,487 individuals from the UK Biobank. The authors were able to identify 203 loci, of which 153 were novel, to be associated with eBMD. These together explained about one third of the total variance in eBMD (17). Although highly successful, none of the previous GWA-studies with DXA-derived BMD have been as successful as the study by Kemp et al.

Despite these great advances, thus far, only <10% of the total estimated genetic variance in BMD can be explained by the results of the performed GWA-studies (18–21). Further, GWASs have predominantly been successful in identifying common variants with a small effect size (Figure 1), which, while giving insight into bone biology, have no clear or direct clinical relevance. However, recent GWASs utilizing whole genome sequencing (WGS) data have been able to identify variants with larger effect sizes. In the largest DXA-derived GWAS to date, Zheng et al. showed that rs11692564, a non-coding SNP around 50 kb downstream of *EN1*, had an estimated effect size of +0.20 SD for lumbar spine BMD (19). Leveraging the Icelandic sequencing initiative with WGS data for >2,000 individuals, rare variants can be imputed and assessed. These data have enabled low frequency variants with large effect sizes for BMD in *COL1A2* and *LGR4* to be identified (22, 23). Several genomic loci, identified through common genetic variation, have also been linked to genes known to underlie monogenic forms of skeletal pathology. In a large meta-analysis on BMD conducted by Estrada et al. (18), the authors were able to identify 60 genes likely to underlie the association signals. Of these, 13 genes (22%) had been implicated in monogenic skeletal disorders and 27 genes (45%) had a corresponding knockout mouse with a skeletal phenotype (14, 18). This demonstrates that even though the signals picked up by GWASs might indicate a weak effect from the measured variation, it is likely that rare and more damaging genetic variations in the same genomic locus might have a large effect. The genomic areas implicated in these GWASs are therefore likely to be of greater importance than the individual signal divulges (24).

While considering the great success of GWASs, the results need to be interpreted in light of the studied trait. Fracture is the most clinically relevant outcome measured, while BMD represents perhaps the best proxy as it is still considered the main determinant for bone strength, and the main diagnostic measurement for osteoporosis (10, 25). BMD measured by quantitative ultrasound (QUS) of the heel (eBMD) can be used as a cost-effective alternative for BMD and is also independently associated with fractures (ISCD Official positions, 2015). The correlation between BMD and eBMD is, however, not very strong (17, 26). Even the DXA-derived BMD is a blunt measurement for bone health and fracture prediction and needs to be considered with other diagnostic parameters when clinically evaluating a patient's skeletal health (27).

### Recent Advances in Genetic Research

As mentioned, several monogenic forms of osteoporosis have been described. Osteogenesis imperfecta (OI) is the best-known form of monogenic osteoporosis and comprises a heterogeneous family of different heritable bone dysplasias with skeletal fragility (28). Parallel to new developments in genetic methodology, new gene discoveries in variable forms of monogenic osteoporosis have been made and, to date, the list of genetic causes of OI and monogenic primary osteoporosis comprises altogether 19 genes (Table 1). The novel genetic findings have considerably enhanced our understanding of the complexities of bone metabolism and uncovered new molecular pathways that regulate bone metabolism and contribute to skeletal pathology. They span beyond the collagen-related pathways to include signaling cascades regulating bone cell function and the extracellular matrix, as described in detail below. The great variability in clinical features and inheritance patterns emphasize the importance of a molecular diagnosis in these patients.

**Table 1:** Different molecular mechanisms and genes underlying osteogenesis imperfecta.

**Pathophysiological mechanism**	**Gene**	**Protein**	**Inheritance**	**Number of known mutations**	**OMIM (Phenotype MIM number)**
Defects in collagen type I synthesis, structure, folding, post-translational modification, processing and cross-linking	*COL1A1*	Collagen alpha-1(I) chain	AD	>1,000*	166200; 166210; 259420; 166220
	*COL1A2*	Collagen alpha-2(I) chain	AD; AR°	>600*	259420; 166210; 166220
	*CRTAP*	Cartilage-associated protein	AR	32*	610682
	*PPIB*	Peptidyl-prolyl cis-trans isomerase B; cyclophilin B	AR	17*	259440
	*P3H1*	Prolyl 3-hydroxylase 1	AR	69*	610915
	*FKBP10*	Peptidyl-prolyl cis-trans isomerase FKBP10	AR	38*	610968
	*PLOD2*	Procollagen-lysine,2-oxoglutarate 5-dioxygenase 2	AR	10*	609220
	*SERPINH1*	Serpin H1	AR	9*	613848
	*BMP1*	Bone morphogenetic protein 1	AR	11*	614856
Defects in other proteins leading to abnormal bone mineralization	*SPARC*	SPARC; osteonectin	AR	2*	616507
	*SERPINF1*	Pigment epithelium-derived factor (PEDF)	AR	38*	613982
	*IFITM5*	Interferon induced transmembrane protein 5	AD	2*	610967
	*PLS3*	Plastin 3	XLD	17	300910
Defects in osteoblast differentiation and function	*TMEM38B*	Trimeric intracellular cation channel type B	AR	6*	615066
	*WNT1*	Proto-oncogene Wnt-1	AR	35*	615220
	*SP7*	Transcription factor Sp7; osterix	AR	2*	613849
	*CREB3L1*	Cyclic AMP-responsive element-binding protein 3-like protein 1	AR	3*	616229
	*MBTPS2*	Membrane-bound transcription factor site-2 protease	XLR	2	301014
Unknown	*TENT5A* (also known as *FAM46A*)	Terminal nucleotidyltransferase 5A	AR	3	617952

AD, autosomal dominant; AR, autosomal recessive; XLD, X-linked dominant; XLR, X-linked recessive.

° Seen only in a few consanguineous families.

* Information taken from the Osteogenesis imperfecta & Ehlers-Danlos syndrome variant databases.

## Paths to Monogenic Osteoporosis

### Defects in Bone Cell Function and Bone Remodeling

**Figure 2:**
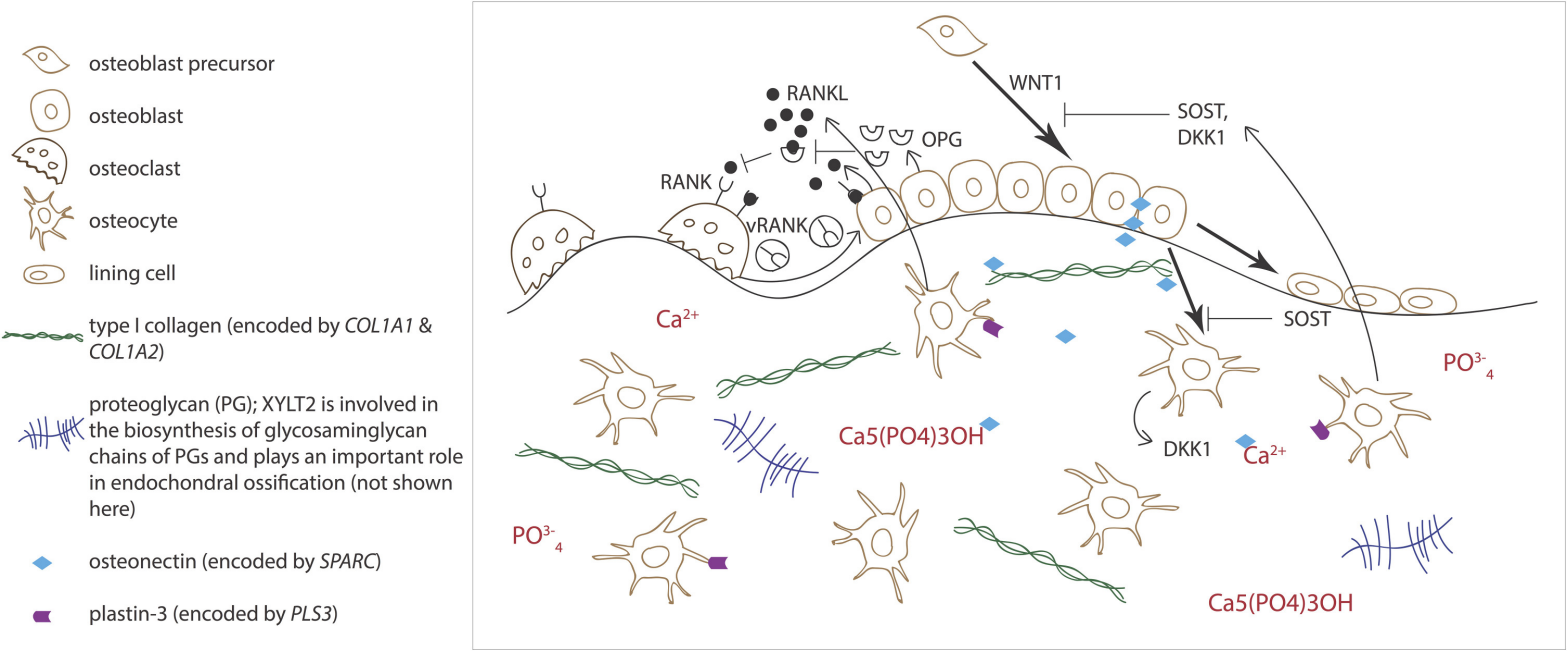
Schematic overview of bone cells and extracellular matrix components involved in regulating bone homeostasis. Receptor activator of nuclear factor kappa-B ligand (RANKL) binds to its conjugate receptor RANK on osteoclast cell surface to stimulate osteoclast differentiation and activity. Osteoprotegerin (OPG) inhibits RANK/RANKL-binding to inhibit bone resorption. WNT signaling pathway stimulates osteoblast function and bone formation. Sclerostin (SOST) and dickkopfs (DKK1), produced by the osteocytes, are two WNT antagonists that promote osteoclasts differentiation. Osteonectin, produced by the osteoblasts, binds calcium, hydroxyapatite and collagen type I and thus regulates bone mineralization. Plastin-3 (PLS3), expressed by the osteocytes, may also be involved in the mineralization of the extracellular matrix but its role in osteoprogenitors and other bone cells is yet to be confirmed.

Normal osteoblast and osteoclast functions are key to sustaining healthy bone tissue. Bone resorption by osteoclasts and formation by osteoblasts are tightly linked in successive repetitive cycles at specific bone sites and the processes are meticulously controlled by several locally produced and circulating systemic factors (29). Communication between the osteoclast and osteoblast is crucial for balanced bone turnover and defects in either cell's function can jeopardize bone health. Osteoblasts express the receptor activator of nuclear factor kappa-B ligand (RANKL), which binds to its conjugate receptor RANK on osteoclast cell surface (Figure 2) (30, 31). This activates osteoclastogenesis and osteoclastic bone resorption. Osteoblasts also secrete osteoprotegerin (OPG) that serves as a decoy receptor for RANKL to inhibit RANKL-RANK–binding, therefore downplaying RANKL's osteoclastogenesis-promoting effect and, as its name implies, protecting bone from over-resorption (Figure 2) (30, 31). Recently, RANK was also noted to relay back by vesicular trafficking from mature osteoclasts to osteoblasts to promote bone formation by reverse signaling (32). The significance of the RANK-RANKL–communication is portrayed in several monogenic conditions with abnormal bone mass resulting from defective RANK-RANKL-OPG–axis: osteoclast-poor osteopetrosis with excessive bone formation due to mutated RANKL, juvenile Paget's disease with osteopenia and progressive skeletal deformity from mutated OPG, and familial expansile osteolysis (FEO) with osteolytic lesions and increased bone remodeling from mutated RANK (33–35).

Alongside osteoblasts and osteoclasts, osteocytes have emerged as key regulators of bone turnover, mineral homeostasis and hematopoiesis (36). Osteocytes are terminally differentiated osteoblasts embedded throughout the mineralized matrix. They communicate with each other and other cells through an extensive network of long cytoplasmic dendritic processes and are thought to orchestrate the interplay between osteoblasts and osteoclasts in bone modeling and remodeling by sensing mechanical loading and responding to endocrine factors, and blood calcium and phosphate concentrations (37). Osteocytes express a range of proteins, such as dentin matric protein 1 (DMP1), phosphate-regulating neutral endopeptidase on chromosome X (PHEX), and matrix extracellular phosphoglycoprotein (MEPE), that are crucial for local matrix mineralization (38). Osteocytes are the primary source of sclerostin, RANKL, and fibroblast growth factor 23 (FGF23), through which osteocytes exert their endocrine functions in bone (Figure 2) (36, 38).

The WNT pathway has a key role in all aspects of bone health—from fetal skeletal development to childhood bone mass accrual to adult bone homeostasis and microarchitectural sustenance (39). WNTs act locally by activating adjacent cells' WNT signaling in a paracrine manner: in developmental stages to partake in the cross-talk between osteoblasts and hematopoietic stem cells (HSCs) in bone marrow and promote bone cell development, differentiation and proliferation, and later in mature adult bone, to induce osteoblastic bone formation (39). WNTs can also act by autocrine means by regulating cells of the same osteoblast or osteoclast lineage (40). The activated pathway is anabolic to bone, leading to increased bone formation and decreased bone resorption. Three different WNT pathways are recognized: the canonical pathway (WNT/β-catenin pathway), the non-canonical planar cell polarity pathway, and the non-canonical WNT/Ca^2+^ pathway. While the latter two, also known as the β-catenin-independent pathways, participate in a range of development process and in bone metabolism, the canonical WNT/β-catenin pathway is considered the predominant pathway maintaining skeletal health (41).

Dysregulated WNT/β-catenin signaling leads to various skeletal disorders of both high and low bone mass. This was first recognized in 2001 when mutations in low-density lipoprotein 5 (*LRP5)*, encoding a coreceptor for WNT ligands, were found to lead to low bone mass in the autosomal recessive osteoporosis pseudoglioma syndrome (OPPG, MIM 259770), characterized by early-onset severe osteoporosis and blindness (42, 43). The *LRP5* mutations inhibit normal WNT signaling and lead to reduced osteoblast proliferation and function and subsequently decreased bone formation (43). Since then, many other mutations in *LRP5* have been shown to cause OPPG (44). In addition, functionally significant SNPs in *LRP5* have been linked to adolescent bone mass accrual and peak bone mass (45, 46), and genome-wide searches have found common *LRP5* polymorphisms that contribute to population-based variance in BMD, confirming its significant role in osteoporosis risk also in the general population (14, 18). The molecular mechanisms by which these missense mutations in *LRP5* decrease WNT signaling, however, remain largely unknown (46, 47). Conversely, inadequate WNT inhibition from mutations or deletions in the sclerostin-encoding *SOST* results in high bone mass phenotypes sclerosteosis (MIM 269500) and van Buchem disease (MIM 239100), respectively (48, 49). In the absence of sufficient sclerostin, WNT signaling is unrestrained, leading to continuous bone formation.

All in all, 19 different WNT proteins are known and together they initiate several intracellular signaling cascades to regulate organogenesis, cell fate determination, primary axis formation, and stem cell renewal (39). Several of the WNT proteins are expressed in bone tissue and regulate bone health at various phases during skeletal growth, development, and e.g., osteoporosis pathogenesis (50). For example, WNT16 is considered an important ligand in bone WNT signaling and has been shown to mediate its bone-specific actions via both canonical and non-canonical WNT pathways (51). Although the specifics behind its mechanisms are unclear, GWASs show that polymorphisms of the *WNT16* locus associate with cortical bone thickness, BMD, and osteoporotic fracture risk in large observational studies and variations in *WNT16* may also impact individual peak bone mass (18, 52, 53). These findings are echoed in *in vivo* studies as *Wnt16* KO mice have reduced cortical thickness and bone strength leading to spontaneous peripheral fractures (54).

**Figure 3:**
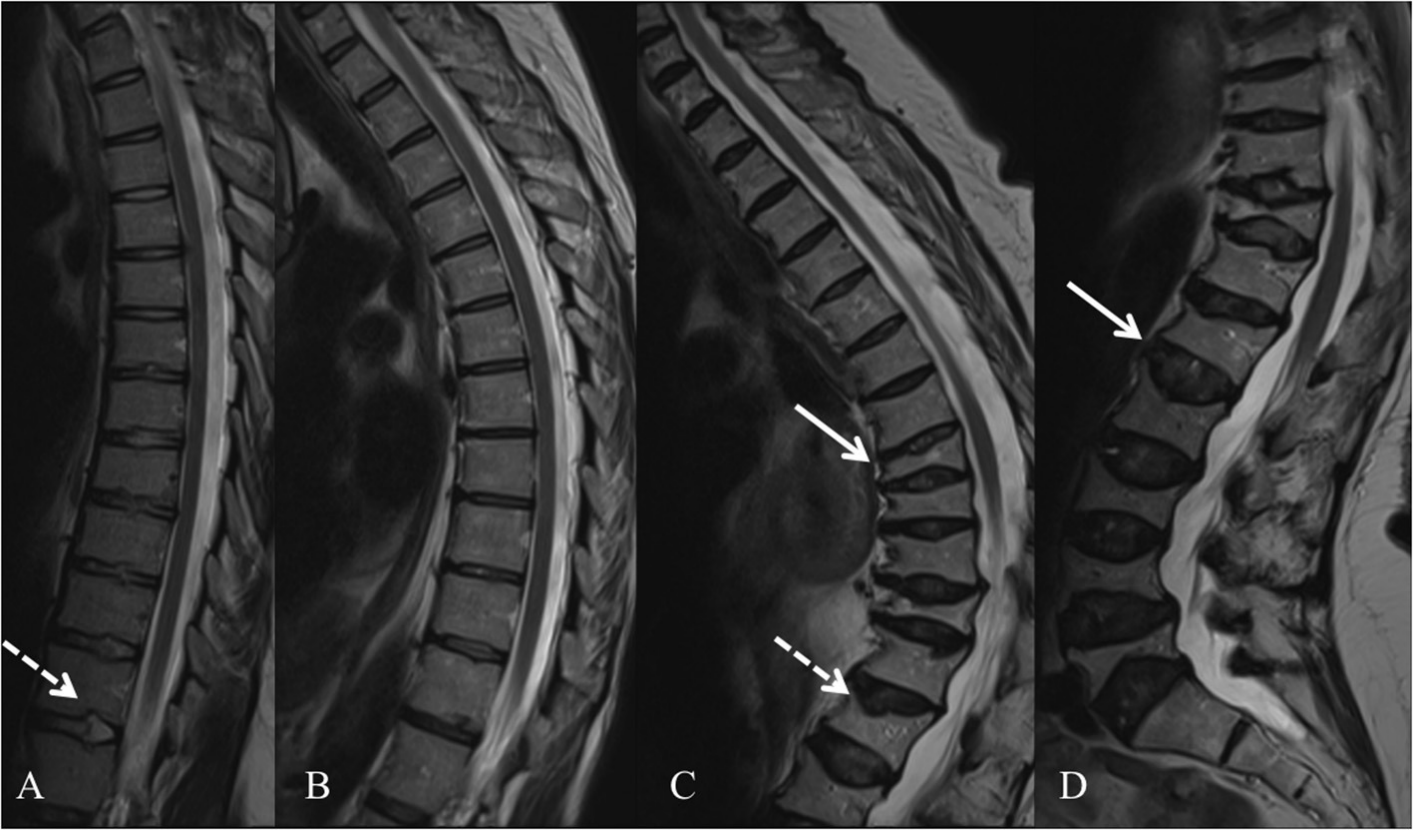
Spinal magnetic resonance images of four *WNT1* p.C218G mutation-positive subjects. **(A)** Thoracic spine of a 17-years-old female showing multiple Schmorl nodes (arrow). **(B)** Thoracic spine of a 44-years-old female showing exaggerated thoracic kyphosis. **(C)** Thoracic spine of a 76-years-old male showing several compressed vertebrae, kyphotic stature, and Schmorl hernia (arrow). **(D)** Lumbar spine of a 74-years-old female showing several compressed vertebrae and enlarged intervertebral discs (arrows). Reprinted from Mäkitie et al. (63) with permission from Elsevier.

In 2013, several groups identified WNT1 as a key ligand to the WNT pathway in bone; heterozygous *WNT1* mutations were reported to cause autosomal dominant osteoporosis, and homozygous mutations, a more severe osteogenesis imperfecta (55). Since then, various other mutations have been found worldwide, all reporting skeletal morbidity with frequent and childhood-onset peripheral and vertebral compression fractures and successive changes in spinal stature (55–61). In our comprehensive clinical analyses of a large cohort of 25 *WNT1* mutation-positive subjects with the same heterozygous missense mutation p.C218G, the aberrant WNT1 signaling results in a severe skeletal pathology (62). In addition to prevalent fractures, long bone modeling is altered and BMD low in affected children, while vertebral compression fractures are very common later in adulthood and result in severe kyphotic deformity and loss of adult height soon after the age of 50 years (Figure 3). Bone biopsy histomorphometry demonstrated low-turnover osteoporosis with scarce and inactive bone cells and stagnant bone turnover. Noted extra-skeletal traits included changes in spinal cartilaginous structures, namely vertebral endplate deterioration and frequent Schmorl nodes, and increased reticulin and early-phase–shifted granulopoiesis as signs of abnormal bone marrow function (63, 64).

The latest finding of dysregulated WNT signaling in monogenic osteoporosis is *SFRP4* mutations in Pyle's disease (65). Frizzled-related protein 4 (SFRP4) acts as an WNT inhibitor and biallelic, truncating mutations in its encoding gene *SFRP4* result in aberrant regulation of WNT signaling, osteoblasts and osteoclast function and bone remodeling (65). The patients' clinical phenotype is predominated by cortical-bone thinning and fragility and expanded metaphyseal trabecular bone, resulting in limb deformity and high propensity to fracture. Correspondingly, *Sfrp4*-null mice present with increased trabecular bone, decreased cortical bone and failure in bone modeling (65).

Despite their important functions, known monogenic forms of bone diseases stemming from osteocyte defects are rare and often relate to defective mineral metabolism, especially hypophosphatemia due to disturbed FGF23 regulation. One of the most recently identified monogenic forms of osteoporosis is caused by mutations in the PLS3 gene (66–70), encoding the actin binding, actin bundling protein plastin 3. This X-linked form of primary early-onset osteoporosis is characterized by low BMD, frequent peripheral fractures and vertebral compression fractures, and subsequent severe thoracic kyphosis. Due to its X-chromosomal inheritance, male patients are more severely affected, usually presenting with severe childhood-onset osteoporosis. Clinical manifestations in females with heterozygous *PLS3* mutations are variable ranging from subclinical osteopenia to a more severe phenotype resembling that of males' (68). The total number of diagnosed patients is still scarce and hence the comprehension of the clinical and genetic spectrum, the disease progression and appropriate treatment is limited.

While the role of PLS3 in bone fragility is yet unknown, one theory presumes PLS3 to alter osteocyte function through abnormal cytoskeletal microarchitecture. Plastins, in general, are Ca-dependent actin binding and bundling proteins and as such, are involved in cytoskeletal arrangements and partake in regulating cellular morphology, motion, and adherence (71). Despite lack of systematic studies, plastin 3 (also called T-plastin) is supposedly expressed in all solid tissues and through indicated functions in other tissues, such as spinal muscle, inner ear stereocilia, and periodontal ligaments, is suggested to be involved in bone mechano-transduction (72–74). This is supported by the high expression of plastin 3 in chicken osteocyte dendrites, especially during dendrite formation (Figure 2) (75–77). Although this is supported by clinical investigations from biochemical and bone biopsy findings indicating that osteocytes appear affected in *PLS3* mutation-positive subjects (78), the observation remains mostly theoretical.

Another suggested role for PLS3 in bone is involvement in mineralization. This is collectively supported by the patients' low BMD and their bone biopsies' histology. We have reported accumulation of non-mineralized osteoid in trabecular bone in patient biopsies (69, 70, 78, 79) and shown that biochemical markers of bone turnover, although not directly echoing the mineralization process, are normal despite altered bone formation (68). The detailed mechanisms of bone tissue mineralization are still debated, but extracellular mineral deposition through budding off of intracellular microvesicles has emerged as one part of the process (80). This process requires dramatic changes in the cell membrane through a complex and well-orchestrated process involving the actin cytoskeleton. Thouverey et al. (81) and Piehl et al. (82) have demonstrated congruently that plastin 3 is involved in the formation of extracellular vesicles. It can thereby be speculated that *PLS3* mutations could have deleterious effects on the mineralization process in bone through defective microvesicle formation, although the details behind this too remain undisclosed.

Lastly, a recent experimental animal study presented new findings suggesting involvement of osteoclast malfunction as part of pathophysiology in PLS3 osteoporosis (83). *In vivo* and *in vitro* studies using *Pls3* knockout and overexpressing mice confirmed the osteoporotic phenotype in the former and thickening cortical bone in the latter. *In vitro* studies of osteoclasts derived from the animals demonstrated a regulatory role of PLS3 in osteoclastogenesis. Additionally, a dysregulation of osteoclast activity was found in cells from *Pls3* knockouts, likely connected to impaired podosome organization due to decreased actin regulation (83). These findings are yet to be confirmed in humans.

### Defects in Bone Extracellular Matrix

In addition to bone cells, reduced bone strength and various skeletal disorders can also stem from defects in the extracellular matrix (ECM). The ECM is primarily composed of different collagenous proteins, non-collagenous proteins (in particular glycoproteins and proteoglycans), lipids, minerals and water (84, 85). The most abundant protein is the type I collagen, made of two alpha-1 and one alpha-2 chains intertwined in a triple helical structure. Mutations in the encoding genes, *COL1A1* and *COL1A2*, respectively, lead to qualitative or quantitative defects in the protein and give rise to osteogenesis imperfecta (OI), a skeletal dysplasia characterized by low BMD and enhanced bone fragility, and often extra-skeletal features, such as blue sclerae, dentinogenesis imperfect, and hearing loss (86, 87). Heterozygous glycine substitutions that affect the Gly-Xaa-Yaa pattern in the triple helix are the most common mutations and can cause mild to lethal OI (87). However, multiexonic deletions or deletion of an entire allele have been sporadically found (88–91). Interestingly, mutations that lead to a reduced amount of normal protein give rise to a milder phenotype than missense mutations affecting the primary structure of the triple helix (dominant negative effect) (87). Furthermore, homozygous glycine substitutions in *COL1A2* have been identified in a handful of consanguineous families (92–95). Surprisingly, the patients harboring biallelic *COL1A2* mutations have a moderate to severe phenotype whereas the mutation carriers are only mildly affected or free from any obvious skeletal impairment. On the other hand, homozygous *COL1A1* mutations are likely to be lethal since they have never been reported in humans. Furthermore, some previous reports have indicated that when the *COL1A1* or *COL1A2* mutation involves the C-propeptide cleavage site, the phenotypic manifestations may include high BMD and mild skeletal fragility (96). A recent study on such cleavage site variants showed that the mutations lead to a distinctive OI phenotype with variable expression, mild to moderate disease severity, moderate fracture rate, high bone mass and increased bone mineral density (97).

Although *COL1A1* or *COL1A2* mutations are detected in ~85% of OI cases, to date, mutations in altogether 17 other genes are also known to cause OI-like skeletal disorders (Table 1). Some of these genes play a role in the post-translational modification of type I collagen while some are key regulators of osteoblast differentiation and function and/or lead to abnormal bone mineralization (Table 1). One example of severe autosomal recessive OI caused by a mineralization defect is linked to mutations in *SPARC* (98). The encoded protein Secreted Protein Acidic and Cysteine Rich, better known as osteonectin, is a glycoprotein that is mainly expressed by osteoblasts during bone formation and binds calcium, hydroxyapatite and collagen type I and other proteins in the ECM (Figure 2). Null mutations in *SPARC* lead to reduced accumulation of type I collagen in the ECM (99). Furthermore, the osteonectin-type I collagen complex is suggested to sequestrate calcium and phosphate in order to initiate bone mineralization (100). An impairment of two other proteins expressed by the osteoblasts, the pigment epithelium-derived factor (encoded by *SERPINF1*) and the interferon-induced transmembrane protein 5 (encoded by *IFITM5*), respectively, can also compromise bone mineralization and lead to OI (86, 87, 101, 102). Most recently, mutations in *FAM46A*, encoding the terminal nucleotidyltransferase 5A, have been detected in four patients with OI. However, the molecular function of this protein and the pathophysiological mechanism by which the mutations lead to OI are not yet known (103).

Besides OI, there are several other skeletal syndromes that feature osteoporosis and are caused by defects in the ECM. For example, mutations in *XYLT2* lead to spondyloocular syndrome characterized by childhood-onset osteoporosis, cataract, cardiac defects and hearing impairment (104–106). The mutated protein xylosyltransferase 2 is involved in the biosynthesis of glycosaminoglycan chains and plays an important role in endochondral ossification and chondrocyte differentiation and maturation. Proteoglycans are also important for other tissues and organs, including brain, heart, and retina, which could explain why the clinical manifestations of spondyloocular syndrome are not only restricted to the skeleton (106).

In addition to causing autosomal recessive OI, inadequate folding and post-translational modification of type I collagen can result in another skeletal syndrome characterized by congenital contractures, named Bruck syndrome. Homozygous mutations in *FKBP10* and *PLOD2* result in Bruck syndrome 1 and 2, respectively (107–110). *FKBP10* encodes the immunophilin FKBP65, a molecular chaperon of type I collagen and *PLOD2* encodes the procollagen-lysine, 2-oxoglutarate 5-dioxygenase 2, which catalyzes the hydroxylation of lysyl residues in type I collagen. Mutations in both *FKBP10* and *PLOD2* can also cause autosomal recessive OI (Table 1).

## Tools for Diagnosing Monogenic Osteoporosis

### Uncovering the Genetics

As discussed, to make the diagnosis of osteoporosis in children two criteria need to be met; (1) low BMD or BMC (Z-score ≤ −2.0 SD) and (2) a clinically significant fracture history. A vertebral fracture indicates severely compromised bone strength and suffices alone for the diagnosis (12). The diagnosis of primary osteoporosis in children can be made when potential causes of secondary osteoporosis, such as other underlying illnesses or medical treatments, have been excluded (2). Most forms of childhood-onset primary osteoporosis are termed osteogenesis imperfecta, although the diagnosis is vague and merely appoints the disease to belong to a heterogeneous group of skeletal disorders with diverse clinical presentation (86, 87). As indicated earlier, the genetic background of OI is heterogeneous and the phenotypic and genetic variability have complicated OI classification. As of yet, there is no consensus indicating which genotype-phenotype combinations should be classified under the umbrella of OI and which should not. The current classification of OI is based on phenotypic features, but the molecular cause is often the key factor determining clinical prognosis, appropriate treatment approach and recurrence risk in the family, and should therefore be emphasized (28). A molecular diagnosis also facilitates the refinement of future treatment and clinical care protocols (87, 111). Although more than 85% of OI cases can still be traced to pathogenic variants in either of the two collagen type I–coding genes *COL1A1* or *COL1A2* (112, 113), the several other genes identified over the past 12 years in OI or monogenic forms of primary osteoporosis need to be kept in mind (92, 114, 115).

While most clinicians begin by screening *COL1A1* and *COL1A2* possibly in combination with MLPA, proceeding to a full OI gene panel using massive parallel sequencing is recommended (87). A sequencing-based gene panel will not only capture sequence variants but also possible structural variations including larger deletions and duplications. Although the surge of new genetic findings has facilitated interpretation of sequence variants, deep intronic splice variants or splice variants masked as synonymous variants are still difficult to correctly annotate. Transcriptome analysis using RNA sequencing together with DNA sequencing has proven successful in increasing the diagnostic yield and assessing functional impact of variants that are otherwise hard to interpret (116). This, however, requires that the disease in focus has a readily accessible proxy tissue, where the gene expression reflects the expression in the affected tissue. Unfortunately, tissue accessibility is very difficult in bone diseases and the method cost-restricted in clinical settings.

Regarding structural variants, WGS has provided an advantage in assessing structural variants compared to exome sequencing or other capture-based protocols. However, all short-read sequencing technologies have shortcomings in their ability to detect and identify structural variants, and, as concluded by Telenti et al. (117), after sequencing 10 000 human genomes the interpretation of structural variants on an individual level still remains challenging. Older methods to indirectly detect structural variations, such as array-based comparative genomic hybridization (array-CGH), are still applicable in specific cases and can help clinicians in their search for a molecular diagnosis (91).

### Clinical Characterization

Owning to the wide spectrum of genetic causes, the clinical presentation of different OI and primary osteoporosis forms is unsurprisingly miscellaneous (87). The diseases vary in their primary skeletal traits, age-at-onset, natural progression, sensitivity to treatment, and presence and spectrum of extra-skeletal characteristics. Although severely compromised bone strength is usually a unifying finding, the DXA-derived BMD, bone biopsy findings, prevalence and type of fractures, and radiographic findings are inconsistent. The phenotypic severity can vary from mild to severe and disease onset from childhood to early adulthood—at times provoked by pregnancy-related calcium loss. Presentation may vary between patients with different mutations and even between family members with identical mutations (87). Classical OI-related extra-skeletal findings include blue sclerae, increased joint laxity, dentinogenesis imperfecta and impaired hearing (28, 87, 118). Mutations in proteins affecting the collagen-related pathways all seem to exhibit similar traits; only the severity and array of affected skeletal sites vary. Some typical presentations include popcorn epiphyseal plates in CRTAP, calcifications of interosseous membranes and hyperplastic callus formation in IFITM5, and skull ossification defects in SEC24D-related OI (Table 1) (86, 87, 118). The extra-skeletal manifestations of bone cell-related forms are still incompletely defined; with monoallelic *WNT1* mutations patients have changes in spinal cartilaginous structures (63) and mild abnormalities in bone marrow hematopoiesis and reticulin formation (119), while in biallelic mutations the phenotype is more severe and OI-like but no bone marrow defects have been reported (55). However, central nervous system manifestations have been reported in some patients with homozygous *WNT1* mutations (55, 61). Patients with *PLS3* mutations do not exhibit any apparent extra-skeletal traits, though this is still scantily explored.

### Novel Biomarkers

In addition to DXA and plain radiography, several factors can be measured from systemic circulation and urine when diagnosing and monitoring patients' disease state, progression and treatment response. The conventional metabolic markers reflect bone turnover and consist of enzymatic and proteinaceous by-products; the most widely used resorption markers include mainly by-products of collagen breakdown, [urinary collagen type 1 cross-linked N-telopeptide (NTX), urinary/serum collagen type 1 cross-linked C-telopeptide (CTX), and collagen fragments from matrix-metalloproteases (ICTP)], and formation markers procollagens from collagen synthesis [serum amino-terminal propeptide (PINP) and carboxyl-terminal propeptide (PICP)] or osteoblast-related proteins (serum osteocalcin (OC) and serum bone isoenzyme of alkaline phosphatase (ALP) (120, 121). While these markers are commonly used and easily analyzed in automated routine laboratories, they do lack specificity and are easily confounded by other patient-related (e.g., body adiposity, inflammation, blood glucose level, time of sampling) and analytical factors. Furthermore, they often respond inadequately to bisphosphonate treatment and correlate poorly with BMD and bone histomorphometric parameters (55, 120–125). None of the monogenic forms of osteoporosis have a specific biomarker profile and these conventional markers are of little value in differentiating between the various genetic forms of osteoporosis.

The limitations of the conventional bone markers have fueled a field-wide search for new potential biomarkers. Zooming into smaller cell-released particles, small microRNAs (miRNAs), as one, have attained much attention and are proposed to hold promise in future diagnostic and treatment in skeletal disorders. These small, non-coding fragments of RNA are highly conserved and comprise, on estimate, 1% of our genome (126, 127). They alter gene expression by RNA silencing and post-transcriptional regulation; each miRNA is predicted to regulate hundreds of different target genes, thus serving important functions in many tissues and biological processes (127, 128). While their exact function in gene regulation is still largely unknown, miRNAs are thought to mediate intercellular communications in various metabolic processes and diseases and a unique imprint of differentially expressed miRNAs is observed in e.g., certain cancers, metabolic diseases and viral infections. In bone, miRNAs contribute to homeostasis and their dysfunctional expression relays to progression of skeletal disorders (129, 130). Their expressions change in result of low BMD, frequent fractures, or menopausal osteoporosis (129, 130).

These findings have encouraged researchers to explore the clinical potential of miRNAs in disease diagnostics and follow-up. Several clinical studies have evaluated miRNA expression in osteoporotic patients and distinguished specific miRNAs correlating with the degree of osteoporosis (131). miR-133a was significantly elevated in postmenopausal Caucasian women with low BMD (132), and miR-194-5p and miR-21-5p negatively correlated with BMD in Chinese osteoporotic women (133, 134). Seeliger et al. (135) also identified miR-21-5p, in addition to four other miRNAs (miR-23a-3p, miR-24-3p, miR-100-5p, and miR-125b-5p) to be differentially expressed in serum and upregulated in bone tissue in patients with osteoporotic fractures. *In vitro* studies have observed miRNAs that interact with known key regulators of bone metabolism, such as miR-152-3p and miR-335-3p with Dickkopf-1 (136, 137), miR-30e-5p with Lrp6 (138), and the aforementioned miR-133 with Runx2 (139). Furthermore, Anastasilakis et al. (140) reported that serum levels of miRNAs changed in response to anti-osteoporotic treatment. While different studies pinpoint to varying miRNAs depending on cohort size, demographic or other factors, a clear congruency is echoed that a unique miRNA signature is observed in osteoporosis.

We have reported altered miRNA pattern in patients with WNT1 osteoporosis, with two upregulated and six downregulated miRNAs, as compared with age and sex-matched mutation-negative controls from the same family (119). While specific miRNA alterations may be recognized in certain monogenic forms of osteoporosis, the role of miRNAs in complementing or substituting genetic testing remains to be explored in future studies. Further, the utilization of miRNA assessments in clinical practice demands further methodological development but based on present data, they hold great potential for future diagnosis and follow-up, including monogenic forms of osteoporosis.

## Options for Treatment

### Conventional Osteoporosis Drugs and Implications for Treatment

Conventional osteoporosis drugs, namely bisphosphonates, have been the mainstay of pharmacological treatment in classical, type I collagen-related OI forms. These typically have high bone turnover and thus the osteoclast-targeting and resorption-decreasing bisphosphonates have proven effective in increasing BMD, reducing fractures, and improving VCFs in patients (141–143). Contrary to collagen I-related OI, bisphosphonates have proven insufficient in improving BMD or fracture tendency in several new forms of primary osteoporosis (55, 57, 60). These OI forms often present with low-turnover osteoporosis and hence the benefits of anti-catabolic treatment are not optimal. We have also shown that patients with prior bisphosphonate treatment have abnormal and apoptotic osteocytes, suggesting adverse effects of bisphosphonates in WNT1 osteoporosis (63). However, our longitudinal study on the effects of teriparatide-treatment in WNT1 osteoporosis indicated that exogenous PTH may be efficient in increasing bone formation and BMD during a 24-months-long treatment in adults; however, there may be simultaneous increase in bone marrow adiposity (79). Thus far, the efficacy of anti-sclerostin antibodies have been experimented in mice only; subcutaneous administration of Scl-Ab to the murine model of WNT1 OI *Wnt1*^*sw*/*sw*^ mice significantly improved fracture rate and increased bone mass that seemed to result from increased osteoblast activity (144).

Besides WNT1-related skeletal pathologies, even less is known about the optimal treatments in other new forms of primary osteoporosis and OI, such as PLS3 and XYLT2 (105, 145). Efficacy of bisphosphonates in PLS3 osteoporosis has been evaluated in a handful of cases and indicate positive response (66, 67, 70). Our above-mentioned clinical study on teriparatide also included *PLS3* mutation-positive subjects and they showed congruent, although slightly lesser, improvement in bone parameters in 24-months follow-up, as compared with patients with WNT1 osteoporosis (79). Patients with *XYLT2* mutations seem to benefit from pamidronate treatment with increase in BMD and improvement in vertebral morphology (104, 105).

Clinical care of OI patients, including both classical and newer forms of OI and monogenic osteoporosis, is often complex and challenging. Means of treatment and pace of clinical follow-up are dependent on the patient's age, clinical manifestations, and degree of impairment, and should be individually tailored and regularly evaluated. Bisphosphonates are still the main treatment option for pediatric patients and are often used to prevent greater decrease in BMD and enable maximum yield in bone mineral throughout childhood and adolescent bone mass accrual. The overall benefits of bisphosphonate treatment in most cases of OI are non-negligible (146). Variable treatment protocols exist. Clinical care and follow-up are advised to be centered in special health care units with abilities to provide multidisciplinary care and expertise.

### Novel Target-Drugs

Discoveries through rare, monogenic forms of skeletal disorders have provided new information on the biology of bone health and revealed previously unidentified proteins that take part in key regulatory pathways. Naturally, these proteins also present as appealing target molecules for development of new treatment modalities. In early 2000s, inhibition of RANKL by a monoclonal antibody denosumab brought a novel approach for treatment of osteoporosis (147). The drug has been used to improve skeletal health in some forms of OI. Particularly patients with *SERPINF1* mutations show a modest increase in BMD in response to denosumab whereas treatment outcomes with bisphosphonates are poor (148). Due to the coupled nature of osteoblast-osteoclast–activity, blocking osteoclastogenesis through RANKL is also unfavorably accompanied by reduced osteoblast function. The previously mentioned discovery of RANKL reverse signaling could offer a novel solution to avoid this problem (32). Also, inhibition of cathepsin K, an osteoclast-derived lysosomal enzyme, seemed promising due to its coupled bone formation-favoring action, but its development was later discontinued due to increased risk of cardiovascular complications (149). As of recently, the effects of anti-TGF-β antibodies have been studied in *Crtap*^−/−^ and *Col1a1*^*frt*/−^ mice with varying results; while the *Crtap*^−/−^ showed great improvements in bone mass and biochemical qualities, *Col1a1*^*frt*/−^ mice did not show significant changes in bone quality or strength (150).

Along with the discovery of van Buchem disease and sclerosteosis, two human models of sclerostin inhibition, fueled the development of a new anabolic target drug named romosozumab—a monoclonal anti-sclerostin antibody targeting the WNT pathway (151, 152). Its efficacy has been evaluated in several clinical trials with promising results; a placebo-controlled, multicenter, phase II study on 419 postmenopausal women with osteoporosis treated with subcutaneous injections of romosozumab at 3-months intervals showed significant, and superior to those attained by alendronate and teriparatide, increase in areal BMD and a tilt in BTMs reflective of increased bone formation (151), and another phase III study reported a reduction in fracture risk in 7,180 postmenopausal osteoporotic women (153). Anti-DKK1 antibodies act similarly to oppose WNT signaling and are potent as osteoanabolic agents. However, administration of anti-DKK1 is only mildly efficacious as the WNT-neutralizing effect is compensated by upregulation of sclerostin, although the opposite is not seen when given only anti-sclerostin antibodies. Thus, the benefits of anti-DKK1 antibodies manifest only when given in conjunction with anti-sclerostin (154).

Another target of interest for new drug development is Notum. It is a secreted enzyme that inhibits WNTs by removing the palmitoleic acid group that is essential for binding of WNTs to Frizzled receptors, thereby inhibiting WNT signaling. Interestingly, experimental studies in rodents have shown that inhibiting Notum through either knockout, or by oral administration of molecular inhibitors or neutralizing antibodies increase cortical bone formation and strength, but do not affect trabecular bone mass (155, 156).

Possible undesired adverse and extra-skeletal effects of new drugs are inevitable as many of the targeted proteins have tissue-wide expression and key roles in various biological processes. Side effects can be latent and subtle but also challenging and life-threatening. Knowing the WNT pathway's fundamental role in embryonic development, tumorigenesis and pathogenesis of other systemic or chronic diseases, romosozumab has been under careful scrutiny for its clinical safety. In mice receiving different doses, no malignancies were noted over a 98-weeks follow up (157). However, along with the robust and positive skeletal effects, use of romosozumab has been associated with cardiovascular and cerebrovascular events, and the drug is currently under FDA review (Amgen and UCB).

### MicroRNAs

Recently, researchers have acknowledged the opportunities in targeting miRNA pathways to develop new therapeutic means and genome editing approaches (128, 158). A few groups have pursued clinical trials to evaluate efficacy of miRNAs in disease target treatment: an on-going clinical trial evaluates the anticancer effect of miRNA lethal-7 in binding to Kirsten rat sarcoma viral oncogene homolog (KRAS) gene in patients suffering from stage III colon cancer, and miR-122 in hepatitis C (159, 160). Bone-specific miRNAs have not been evaluated clinically, but analyses have shown that for example *in vitro* miR-21 could promote osteogenesis in bone marrow stem cells, and systemic administration of miR-214 induced BMD increase and miR-92a enhance fracture healing in mice (161–163). In fracture healing, also angiogenesis is vital to the repair process and Li et al. (164) were able to demonstrate that implantation of MSCs transfected with an angiogenesis-involved anti-miR-26a showed good bone repair. Further, anti-miR-31-transfected MCSs efficiently repaired bone defects by increasing BMD and new bone volume (165). These findings and the efficacy, safety and possible side effects need to be confirmed and carefully evaluated in clinical settings *in vivo*.

## Conclusions

Recent advances in genetic methodology have resulted in several new discoveries relating to the genetic architecture of bone homeostasis. Not only have the basic clinical and genetic pillars of classical OI been refined, but several new forms of monogenic osteoporosis have also been identified that have pinpointed novel molecular mechanisms contributing to skeletal health and disease. The clinical presentation, inheritance mode, natural course and response to conventional osteoporosis drugs are diverse, often variable and logically dependent on the affected protein. Although uncovering the limitations in our current diagnostic and treatment modalities, they have also provided new signaling pathways that hold promise in new targeted drug development. Future research will hopefully continue expanding the genetics and molecular mechanisms behind bone metabolism and increasing our understanding of the specific skeletal and extra-skeletal characteristics of monogenic osteoporosis, while finding new avenues for improved diagnosis and treatment of patients with severe bone diseases.

## Author Contributions

RM and OM initiated the manuscript. RM wrote the first draft. All authors contributed to the writing and approved the final manuscript.

### Conflict of Interest Statement

The authors declare that the research was conducted in the absence of any commercial or financial relationships that could be construed as a potential conflict of interest.
